# A two‐step formula constant optimization strategy for minimal standard deviation and zero mean prediction error in IOL power calculation

**DOI:** 10.1111/aos.17569

**Published:** 2025-08-05

**Authors:** Achim Langenbucher, Nóra Szentmáry, Jascha Wendelstein, Alan Cayless, Peter Hoffmann, Damien Gatinel

**Affiliations:** ^1^ Department of Experimental Ophthalmology Saarland University Saarbrücken Germany; ^2^ Dr. Rolf M. Schwiete Center for Limbal Stem Cell and Aniridia Research Saarland University Saarbrücken Germany; ^3^ Department of Ophthalmology Semmelweis University Budapest Hungary; ^4^ Department of Ophthalmology Ludwig‐Maximilians‐University Munich Germany; ^5^ School of Physical Sciences The Open University Milton Keynes UK; ^6^ Augen‐Und Laserklinik Castrop‐Rauxel Castrop‐Rauxel Germany; ^7^ Rothschild Foundation Hospital Paris France

**Keywords:** IOL power formula, lens constant optimization, nonlinear optimization, refractive outcome, two‐step strategy

## Abstract

**Purpose:**

To investigate the precision and accuracy performance of a two‐step approach for optimizing lens formula constants (FC) with a refractive offset correction (RO) as a second tuning parameter.

**Methods:**

Using IOLMaster 700 biometric data from 4 datasets (886/613/821/467 eyes treated with the Hoya Vivinex/Johnson&Johnson ZCB00/Alcon SA60AT/Bausch&Lomb MX60 lens), and the power of the implanted lens and postoperative spherical equivalent refraction, FC and RO were optimized for SRKT, Hoffer Q, Holladay 1, and Haigis formulae using an iterative nonlinear optimization for RMSPE and sequentially according to Gatinel, optimizing first FC for standard deviation prediction error (SDPE) and then RO by the resulting mean prediction error.

**Results:**

The simple two‐step approach yielded comparable results for the FC and RO for all four formulae and datasets under test. The differences in the formula prediction error were commonly in the third decimals comparing both optimization strategies without clinical relevance. Direct optimization of the FC for SDPE showed large offsets in the formula constant for all datasets and especially for the Hoffer Q and Haigis formulae, resulting in systematic refractive offset values in the formula prediction.

**Conclusions:**

This simple two‐step approach using FC with a second tuning parameter performs excellently for the four formulae and four datasets under test and allows for both reducing the scatter and zeroing the refractive offset. Multicentric studies with other populations and biometers are required to further investigate the clinical applicability.

## 1 BACKGROUND

There is currently no consensus on how to optimize intraocular lens (IOL) formula constants in a clinical setting (Langenbucher et al., 2022; Langenbucher, Szentmáry, Cayless, Müller, et al., 2021). For fully disclosed formulae with a single formula constant (FC) (e.g. third‐generation formulae such as SRK/T; Retzlaff et al., 1990; Sanders et al., 1990), Hoffer Q (Hoffer, 1980, 1981, 1993), Holladay 1 (Holladay et al., 1988) or 4th generation formulae (e.g. simplified Haigis formula (Haigis et al., 2000)) many researchers use formula inversion to solve the formula for the FC (Aristodemou et al., 2011; Langenbucher et al., 2022; Langenbucher, Szentmáry, Cayless, Müller, et al., 2021). For a dataset with *N* subjects, we generate *N* individual FC values, and the mean or median of the distribution of these individual FC values could be used as the optimized formula constant (Shrivastava et al., 2021). However, this strategy does not optimize the FC for any metric of the formula prediction error (PE), defined as the difference between the achieved spherical equivalent refraction after cataract surgery and the formula predicted spherical equivalent refraction (Langenbucher et al., 2022; Langenbucher, Szentmáry, Cayless, Müller, et al., 2021).

In Gatinel, Debellemanière, Saad, Rampat, et al. (2023) published a paper describing a simple concept for optimizing the FC in terms of zeroing the mean PE (MPE) or minimizing the standard deviation (SDPE) or root mean squared prediction error (RMSPE) (Gatinel, Debellmaniére, Saad, Rampat, et al., 2024). This concept uses a start value for the formula constant with the respective PE together with the mean corneal power value and mean IOL power value of the dataset. The correction of the FC is derived using a gradient descent method, and we found that in most situations a single iteration is sufficient to determine the optimized FC in a clinical setting (Langenbucher et al., 2024, 2025).

However, most of the classical IOL power formulae use a modulation of the ‘effective IOL position’ (ELP) to tune the formula for different IOL types or clinical settings (Norrby & Koranyi, 1997; Olsen, 2006; Olsen & Hoffmann, 2014). This ELP value refers to a fictitious axial position of a thin IOL with respect to the corneal front apex and is not necessarily related to the actual anatomical position of the IOL in the pseudophakic eye (Langenbucher, Szentmáry, Cayless, Müller, et al., 2021; Langenbucher, Szentmáry, Cayless, Weisensee, et al., 2021), especially in cases where the corneal power is misinterpreted by the use of an inappropriate keratometer index (nK) from the geometrical data of the cornea. For short eyes (Zhang et al., 2019) requiring high powered IOLs a slight change in ELP has a significant impact on the predicted spherical equivalent refraction (SEQ) at the spectacle plane, whereas in long eyes requiring low powered IOLs the impact of an axial shift of the IOL is much less (or even reversed for negatively powered IOL).

In formula constant optimization, we should strictly differentiate between precision and accuracy of the refractive outcome. Precision refers to the (stochastic) scatter of the refraction irrespective of the MPE being close to zero or showing a systematic offset (Gatinel, Debellemanière, Saad, Wallerstein, et al., 2023). Accuracy relates not to the data scatter itself, but to the deviation of the PE values from the target value of zero. As the scatter in PE (e.g. SDPE) and the MPE are not linked, optimizing the FC for zero MPE does not necessarily minimize the SPDE, and vice versa, minimizing the FC for lowest SDPE does not necessarily zero the MPE (Gatinel, Debellemanière, Saad, Wallerstein, et al., 2023; Langenbucher, Hoffmann, et al., 2023; Langenbucher, Szentmáry, et al., 2023). This is the main reason why, for example, in www.IOLCon.org we use the root mean squared formula prediction error for optimisation of formula constants, as this metric includes both the MPE and the SDPE.

It therefore seems to be a logical step to utilize a second formula constant to optimize the refractive outcome for both precision and accuracy. Some modern formulae such as the Castrop formula already implement additional formula constants independent from the classical ELP shift to tune both precision and accuracy (Langenbucher, Szentmáry, Cayless, Weisensee, et al., 2021). In the formula constant triplet C/H/R of the Castrop formula, the C and the H constants are related to a shift of the ELP whereas the R constant refers to a shift of SEQ which, for example, considers the lane distance in refractometry. In a recent paper published in Gatinel, Debellemanière, Saad, Brenner, et al. (2024) described a two‐step procedure where the classical formula constant is first tuned to minimize the SDPE (precision), and then in a second step the resulting MPE derived with the FC from step 1 is zeroed by simply adding an offset value to the predicted refraction (RO) for the entire dataset. Consequently, since the RO value may differ for different IOL models, we have to deal with two formula constants: the classical constant tuning the ELP and the RO which shifts the predicted refraction (Gatinel, Debellemanière, Saad, Rampat, et al., 2024).

The purpose of this study is

- To show the applicability and to retrospectively evaluate the performance of the two‐step formula constant optimization strategy using a FC and an RO as described by Gatinel, Debellmaniére, Saad, Rampat, et al. (2024) with some clean datasets relating to modern IOL models.
- To compare the results of this two‐step formula constant optimization strategy to an up‐to‐date iterative linear optimization technique based on either zeroing the MPE or minimizing the SDPE with the FC.
- To compare the results to an iterative linear optimization strategy which simultaneously optimizes the FC and RO to minimize RMSPE.

## 2 METHODS

### 2.1 Datasets for our study

Four datasets were analysed in this retrospective study. The first dataset contains measurements from 886 eyes (488 right and 396 left eyes) treated with the 1‐piece hydrophobic aspherical (aberration correcting) monofocal intraocular Vivinex IOL (Hoya Surgical, Singapore). The second dataset contains measurements from 613 eyes (314 right and 299 left eyes) treated with the 1‐piece hydrophobic aspherical (aberration correcting) monofocal intraocular ZCB00 IOL (Johnson & Johnson Vision, Jacksonville, USA). The third dataset contains measurements from 821 eyes (415 right and 406 left eyes) treated with the 1‐piece hydrophobic spherical monofocal intraocular SA60AT IOL (Alcon, Fort Worth, USA). The fourth dataset contains measurements from 467 eyes (278 right and 189 left eyes) treated with the 1‐piece hydrophobic aspherical (aberration free) monofocal intraocular MX60 IOL (Bausch & Lomb, Rochester, USA).

All of the datasets supplied to us contained data from only 1 eye per patient. Where data from both eyes were available, 1 eye was randomly selected for inclusion in the dataset prior to transfer to us. Data from eyes with a history of ocular surgery (other than cataract surgery) or any ocular pathology which could potentially affect the validity of the refractive result after cataract surgery (e.g. ectatic diseases or retinal pathologies) were discarded from the dataset at the source.

All eyes underwent cataract surgery at the Augen‐ und Laserklinik Castrop‐Rauxel, Castrop‐Rauxel, Germany. The local Institutional Review Board (Ärztekammer des Saarlandes, registration number 157/21) provided a waiver for this study, and patient informed consent was not required for this study. The data were transferred to us in an anonymized fashion, precluding back‐tracing of the patient.

The anonymized datasets contained preoperative biometric data from the IOLMaster 700 (Carl‐Zeiss‐Meditec, Jena, Germany), including: axial length AL, anterior chamber depth ACD measured from the corneal epithelium to the anterior apex of the crystalline IOL, the central thickness of the crystalline IOL LT, the corneal front surface radii measured in the flat and steep meridians R1 and R2, the labelled refractive power of the intraocular IOL IOLP, and the spherical equivalent of manual subjective refraction as documented 4 to 12 weeks after cataract surgery by an experienced optometrist at a refraction lane distance of 6 m. To ensure the reliability of the postoperative refraction, the dataset included only data from uneventful cataract surgeries without a previous history of eye surgeries and with a postoperative Snellen decimal visual acuity of 0.8 (20/25 Snellen lines) or higher (Langenbucher, Szentmáry, Cayless, Müller, et al., 2021; Langenbucher et al., 2022).

### 2.2 Preprocessing of the data

The anonymized Excel data (.xlsx‐format) were imported into MATLAB (Matlab 2022b, MathWorks, Natick, USA) for further processing with a custom data processing code. The mean corneal radius R was derived as the harmonic mean of the radii of curvature in the flat and steep meridians, and the mean keratometric power K was calculated as the arithmetic mean of the keratometric power as converted from the corneal radii in both cardinal meridians using a keratometer index as indicated in the respective IOL power formulae.

The following IOL power calculation formulae were considered in this constant optimization process:

- SRK/T formula published by Sanders, Retzlaff, and Kraff (Retzlaff et al., 1990; Sanders et al., 1990).
- Hoffer Q formula published by Hoffer (Hoffer, 1980, 1981, 1993).
- Holladay 1 formula published by Holladay and Prager (Holladay et al., 1988).
- Haigis formula in the simplified version (Haigis et al., 2000).

The SRKT, HofferQ, and Holladay 1 formulae consider the AL and R or K data together with one formula constant (FC: A, pACD, and SF, respectively). The Haigis formula considers the AL, ACD, and R together with a formula constant triplet a0/a1/a2. As the strategy of IOL formula constant optimization described by Gatinel, Debellemanière, Saad, Rampat, et al. (2023) and Gatinel, Debellemanière, Saad, Wallerstein, et al. (2023) (Gatinel, Debellmaniére, Saad, Rampat, et al., 2024) is restricted to single constant formulae, we used a form of the Haigis formula with preset values for a1/a2 = 0.4/0.1 and optimized for a0 only.

For all four formulae under test, we first derived the optimized formula constants using the iterative nonlinear sequential quadratic programming algorithm (SQP) as described in previous papers (Langenbucher et al., 2022, 2024, 2025; Langenbucher, Hoffmann, et al., 2023; Langenbucher, Szentmáry, et al., 2023; Langenbucher, Szentmáry, Cayless, Müller, et al., 2021; Langenbucher, Szentmáry, Cayless, Weisensee, et al., 2021) with the formula prediction error PE (defined as the difference between the formula prediction and the achieved postoperative SEQ) as the target parameter. As a reference, FC optimization was performed for zero MPE and minimal SDPE and RMSPE. In addition, we simultaneously optimized FC and RO, and FC and nK using the SQP algorithm, both for minimal RMSPE. A step size tolerance of 1e‐10 and a function tolerance of 1e‐12 were used as the stopping criteria for the algorithm. In the final step, we used the two‐step sequential approach described by Gatinel, Debellemanière, Saad, Brenner, et al. (2024) in which the first step optimizes FC for minimal SDPE and the second step calculates the refractive offset correction RO for zero MPE. This two‐step optimization was restricted to 1 iteration only (Langenbucher et al., 2024, 2025) as we feel that in a clinical setting multiple iterations requiring calculation of an updated FC or RO and an updated formula predicted refraction might be too complex. The performance metrics MPE, SDPE and RMSPE were derived for all optimization strategies and for all four datasets.

### 2.3 Statistical analysis and data presentation

Data are listed descriptively in terms of the arithmetic mean, standard deviation (SD), median, and the lower and upper boundaries of the 95% confidence interval (2.5% and 97.5% quantiles). The values for the optimized FC, RO (where appropriate) and nK (where appropriate) and the corresponding outcome data in terms of MPE, SDPE, and RMSPE are listed for all formulae under test and all optimizations.

## 3 RESULTS

Table 1 lists the descriptive data for the preoperative biometric measures together with the labelled IOL power and the spherical equivalent of postoperative refraction for the four clinical datasets considered in our data analysis.

**TABLE 1:** Descriptive statistics of the 4 datasets in terms of mean, standard deviation (SD), median, and the lower (quantile 2.5%) and upper (quantile 97.5%) boundaries of the 95% confidence interval.

Explorative description		AL in mm	ACD in mm	LT mm	R12 in mm	K12 in D	PIOL in D	SEQ in D
Dataset 1: Hoya Vivinex lens (*N* = 886)	Mean	24.0922	3.1848	4.6215	7.7641	43.5207	20.6377	−0.5624
	SD	1.4034	0.4072	0.4499	0.2681	1.5012	3.7215	0.9246
	Median	23.9001	3.1847	4.5932	7.7626	43.4777	21.0000	−0.2500
	Quantile 2.5%	21.6749	2.3715	3.7563	7.2686	40.6564	12.0000	−2.5000
	Quantile 97.5%	27.3536	3.9439	5.5194	8.3013	46.4329	27.5000	0.5000
Dataset 2: Johnson & Johnson ZCB00 lens (*N* = 613)	Mean	23.4558	3.1755	4.6388	7.6729	4.0375	22.3418	−0.5137
	SD	1.3958	0.4084	0.4235	0.2636	1.5056	3.9449	0.7897
	Median	23.3800	3.1900	4.6400	7.6699	44.0033	22.0000	−0.2500
	Quantile 2.5%	20.7713	2.3350	3.7100	7.1747	41.0542	14.0000	−2.5000
	Quantile 97.5%	26.9535	3.9817	5.4487	8.2209	47.0402	31.0875	0.5000
Dataset 3: Alcon SA60AT lens (*N* = 821)	Mean	23.1467	3.0434	4.6219	7.6977	43.8971	22.7369	−0.4780
	SD	1.5107	0.3986	0.4120	0.2656	1.5355	4.5956	0.7152
	Median	23.1800	3.0260	4.6100	7.7297	43.6629	22.5000	−0.2500
	Quantile 2.5%	20.4510	2.3060	3.8200	7.1052	41.2601	13.5000	−2.6250
	Quantile 97.5%	26.4297	3.8180	5.4200	8.1898	47.5006	33.0000	0.5000
Dataset 4: Bausch & Lomb MX60 lens (*N* = 467)	Mean	24.4409	3.2443	4.6362	7.7424	43.6336	19.8340	−0.7586
	SD	2.0697	0.3521	0.3895	0.2431	1.3575	5.3005	0.8855
	Median	24.0400	3.2600	4.6600	7.7290	43.6670	21.0000	−0.5000
	Quantile 2.5%	21.4387	2.5859	3.8817	7.3435	41.1209	4.0000	−2.7500
	Quantile 97.5%	31.3795	3.9440	5.4195	8.2075	45.9590	29.0000	0.2500

*Note*: Parameters listed are: axial length (AL), external phakic anterior chamber depth measured from the corneal front apex to the front apex of the crystalline lens (ACD), central lens thickness (LT), harmonic mean of corneal radii in the flat and steep meridian (R12), corneal power converted from R12 with the Javal keratometer index nK = 1.3375 (K12), refractive power of the intraocular lens implant (PIOL) and the spherical equivalent power achieved 4–12 weeks after cataract surgery (SEQ).

Table 2 summarizes the optimized FC (and RO/nK values) for the 4 datasets and for the SRK/T, Hoffer Q, Holladay 1, and Haigis formulae as derived with the following optimization metrics: zeroing MPE with FC (a), minimizing SDPE with FC (b) and RMSPE with FC (c), minimizing RMSPE with FC/RO (d) and FC/nK (e), and the two‐step approach minimizing SDPE with FC and MPE with RO (f). From the table, we see that the results of optimizing FC for zero MPE (a) or minimal RMSPE (c) are quite similar, but optimizing FC for minimal SDPE (b) yields FC values which could be far off. The data also indicate that there is no clinically relevant difference between the simultaneous optimizations for FC/RO (d) and FC/nK (e), and both match very well to the two‐step approach of Gatinel (f).

**TABLE 2:** Formula constants (FC, shaded in light grey) optimizations for zero mean prediction error (MPE, a), minimal standard deviation of prediction error (SDPE, b), minimal root mean squared prediction error RMSPE (c), simultaneous optimization of FC and refractive offset correction (RO, shaded in light green) (d) and FC and the keratometer index (nK, shaded in light blue) (e) for minimal RMSE, and the twe‐step optimization strategy (f) described by Gatinel, Debellemanière, Saad, Brenner, et al. (2024) with FC optimization for SDPE in a first step and RO optimization for MPE in a second step. In this table, four different datasets and four classical third and fourth generation formulae (SRK/T), Hoffer Q, Holladay 1, and simplified Haigis formula were considered. Comparing (d) and (f) shows the slight differences between the direct nonlinear iterative optimization for minimal root mean squared prediction error with FC and R0 and the two‐step optimization procedure (minimizing SD with FC and zeroing MPE with R0).

Dataset	Optimization strategy ➔	Nonlinear iterative optimization techniques							two‐step optimization (Gatinel)	
	Optimization metric ➔	MPE (a)	SDPE (b)	RMSPE (c)	RMSPE (d)		RMSPE (e)		SDPE: FC; MPE: RO (f)	
	Optimization target ➔	FC	FC	FC	FC	RO in D	FC	nK	FC	RO in D
Dataset 1: Hoya Vivinex lens (*N* = 886)	SRK/T	119.2688	119.3868	119.2742	119.3868	−0.0962	119.5086	1.3345	119.3728	−0.0848
	Hoffer Q	5.7631	5.1551	5.7352	5.1551	0.8126	5.1963	1.3312	5.1328	0.8430
	Holladay 1	1.9819	1.6710	1.9676	1.6710	0.4190	1.7073	1.3306	1.6321	0.4607
	Haigis	1.5880	1.0206	1.5631	1.0206	0.7694	1.0081	1.3255	0.9889	0.8130
Dataset 2: Johnson & Johnson ZCB00 lens (*N* = 613)	SRK/T	119.4441	119.7860	119.4606	119.7860	−0.3065	119.8495	1.3358	119.7969	−0.3162
	Hoffer Q	5.8551	5.1928	5.8239	5.1928	0.9821	5.2511	1.3301	5.1650	1.0244
	Holladay 1	2.0874	1.8673	2.0768	1.8673	0.3195	1.8808	1.3311	1.8387	0.3613
	Haigis	1.6507	0.9234	1.6181	0.9830	1.0001	0.9147	1.3232	0.8798	1.1631
Dataset 3: Alcon SA60AT lens (*N* = 821)	SRK/T	118.8833	119.4649	118.9199	119.4649	−0.5425	119.5132	1.3375	119.5172	−0.5907
	Hoffer Q	5.4364	4.9902	5.4091	4.9902	0.6879	5.0230	1.3322	4.9568	0.7401
	Holladay 1	1.7037	1.6496	1.7003	1.6496	0.0817	1.6543	1.3328	1.6302	0.1110
	Haigis	1.2382	0.7395	1.2090	0.7395	0.7795	0.7294	1.3255	0.7006	1.3315
Dataset 4: Bausch & Lomb MX60 lens (*N* = 467)	SRK/T	119.2534	119.3017	119.2574	119.3017	−0.0380	119.3444	1.3336	119.3159	−0.0491
	Hoffer Q	5.8057	5.1339	5.7499	5.1339	0.8637	5.1951	1.3311	5.1103	0.8947
	Holladay 1	2.0016	1.5741	1.9661	1.5741	0.5422	1.6000	1.3295	1.5424	0.5829
	Haigis	1.5887	1.1845	1.5563	1.1845	0.5245	1.1843	1.3245	1.1659	0.5489

Table 3 lists the respective formula prediction error MPE, SDPE, and RMSPE for the 4 datasets and the SRK/T, Hoffer Q, Holladay 1, and Haigis formulae as derived with the FC (and RO / nK) values shown in Table 2. The cells of the table where the formula prediction error metric matched the optimization metric are highlighted in bold. The table shows that MPE, SDPE, and RMSPE obtained from optimizing FC for zero MPE (a) or minimal RMSPE (c) are quite similar, whereas optimizing FC for minimal SDPE (b) could give a large refractive offset (MPE) which is also reflected by the large deviation between SDPE and RMSPE. The SDPE and RMSPE values for the simultaneous optimizations of FC/RO (d), FC/nK (e), and the two‐step approach of Gatinel (f) are quite similar to each other but systematically lower than (a), (b) and (c), especially for the Hoffer Q and Haigis formulae. The data indicate that there is no clinically relevant difference between the simultaneous optimizations for FC/RO (d) and the two‐step approach of Gatinel (f) where FC and RO are optimized sequentially.

**TABLE 3:** Performance of the formula predictions with formula constant (FC), refractive offset values (RO), and tuned keratometer index values (nK) as described in Table 2 in terms of mean prediction error (MPE), standard deviation prediction error (SDPE), and root mean squared prediction error (RMSPE). Optimization was performed for FC for zero mean prediction error (MPE, a), minimal standard deviation of prediction error (SDPE, b), minimal root mean squared prediction error RMSPE (c), simultaneous optimization of FC and refractive offset correction (RO) (d) and FC and the keratometer index (nK) (e) for minimal RMSE, and the two‐step optimization strategy (f) described by Gatinel et al. with FC optimization for SDPE in a first step and RO optimization for MPE in a Gatinel, Debellemanière, Saad, Brenner, et al. (2024).

Dataset	Optimization strategy ➔		Nonlinear iterative optimization techniques					Two‐step optimization (Gatinel)
	Optimization target ➔		FC (a)	FC (b)	FC (c)	FC/RO (d)	FC/nK (e)	SDPE: FC MPE: RO (f)
	Optimization metric ➔		MPE	SDPE	RMSPE	RMSPE	RMSPE	
Dataset 1: Hoya Vivinex lens (*N* = 886)	SRK/T	MPE	**0.0000**	−0.0962	−0.0044	0.0000	0.0050	**0.0000**
		SDPE	0.4414	**0.4409**	0.4413	0.4409	0.4391	**0.4409**
		RMSPE	0.4414	0.4512	**0.4413**	**0.4409**	**0.4391**	0.4409
	Hoffer Q	MPE	**0.0000**	0.8126	0.0367	0.0000	−0.0011	**0.0000**
		SDPE	0.4305	**0.3924**	0.4273	0.3924	0.3892	**0.3924**
		RMSPE	0.4305	0.9024	**0.4288**	**0.3924**	**0.3892**	0.3924
	Holladay 1	MPE	**0.0000**	0.4090	0.0187	0.0000	0.0020	**0.0000**
		SDPE	0.4265	**0.4170**	0.4257	0.4170	0.4187	**0.4171**
		RMSPE	0.4265	0.5841	**0.4261**	**0.4170**	**0.4187**	0.4171
	Haigis	MPE	**0.0000**	0.7694	0.0333	0.0000	−0.0011	**0.0000**
		SDPE	0.4056	**0.3709**	0.4028	0.3709	0.3673	**0.3711**
		RMSPE	0.4056	0.8541	**0.4041**	**0.3709**	**0.3673**	0.3711
Dataset 2: Johnson & Johnson ZCB00 lens (*N* = 613)	SRK/T	MPE	**0.0000**	−0.3065	−0.0149	0.0000	0.0034	**0.0000**
		SDPE	0.4514	**0.4460**	0.4509	0.4460	0.4432	**0.4460**
		RMSPE	0.4514	0.5412	**0.4511**	**0.4460**	**0.4432**	0.4460
	Hoffer Q	MPE	**0.0000**	0.9821	0.0453	0.0000	−0.0014	**0.0000**
		SDPE	0.4629	**0.4090**	0.4583	0.4090	0.4046	**0.4091**
		RMSPE	0.4629	1.0639	**0.4606**	**0.4090**	**0.4046**	0.4091
	Holladay 1	MPE	**0.0000**	0.3195	0.0152	0.0000	0.0006	**0.0000**
		SDPE	0.4326	**0.4267**	0.4321	0.4267	0.4270	**0.4268**
		RMSPE	0.4326	0.5330	**0.4323**	**0.4267**	**0.4270**	0.4268
	Haigis	MPE	**0.0000**	1.0958	0.0481	0.0043	−0.0016	**0.0000**
		SDPE	0.4762	**0.4137**	0.4712	0.4142	0.4072	**0.4139**
		RMSPE	0.4762	1.1713	**0.4736**	**0.4142**	**0.4072**	0.4139
Dataset 3: Alcon SA60AT lens (*N* = 821)	SRK/T	MPE	**0.0000**	−0.5425	−0.0345	0.0000	0.0035	**0.0000**
		SDPE	0.4881	**0.4673**	0.4856	0.4673	0.4625	**0.4674**
		RMSPE	0.4881	0.7160	**0.4868**	**0.4673**	**0.3626**	0.4674
	Hoffer Q	MPE	**0.0000**	0.6879	0.0000	0.0000	−0.0014	**0.0000**
		SDPE	0.4635	**0.4293**	0.4293	0.4293	0.4265	**0.4295**
		RMSPE	0.4635	0.8108	**0.4293**	**0.4293**	**0.4265**	0.4295
	Holladay 1	MPE	**0.0000**	0.0816	0.0051	0.0000	0.0004	**0.0000**
		SDPE	0.4433	**0.4429**	0.4434	0.4429	0.4429	**0.4430**
		RMSPE	0.4433	0.4504	**0.4434**	**0.4429**	**0.4429**	0.4430
	Haigis	MPE	**0.0000**	0.7795	0.0450	0.0000	−0.0012	**0.0000**
		SDPE	0.4610	**0.4184**	0.4564	0.4184	0.4151	**0.4187**
		RMSPE	0.4610	0.8847	**0.4587**	**0.4184**	**0.4151**	0.4187
Dataset 4: Bausch & Lomb MX60 lens (*N* = 467)	SRK/T	MPE	**0.0000**	−0.0380	−0.0032	0.0000	0.0033	**0.0000**
		SDPE	0.4564	**0.4563**	0.4564	0.4563	0.4559	**0.4563**
		RMSPE	0.4564	0.4579	**0.4564**	**0.4563**	**0.4559**	0.4563
	Hoffer Q	MPE	**0.0000**	0.8637	0.0704	0.0000	−0.0016	**0.0000**
		SDPE	0.4882	**0.4146**	0.4775	0.4146	0.4104	**0.4147**
		RMSPE	0.4882	0.9580	**0.4826**	**0.4146**	**0.4104**	0.4147
	Holladay 1	MPE	**0.0000**	0.5422	0.0446	0.0000	0.0012	**0.0000**
		SDPE	0.4538	**0.4237**	0.4492	0.4237	0.4249	**0.4239**
		RMSPE	0.4538	0.6881	**0.4514**	**0.4237**	**0.4249**	0.4239
	Haigis	MPE	**0.0000**	0.5245	0.0415	0.0000	−0.0017	**0.0000**
		SDPE	0.4122	**0.3824**	0.4078	0.3824	0.3795	**0.3825**
		RMSPE	0.4122	0.6491	**0.4099**	**0.3824**	**0.3795**	0.3825

*Note*: In this table, four different datasets and four classical third and fourth generation formulae (SRK/T, Hoffer Q, Holladay 1, and simplified Haigis formula with preset a1 = 0.4 and a2 = 0.1) were considered. Bold values indicate statistically significance.

**FIGURE 1:**
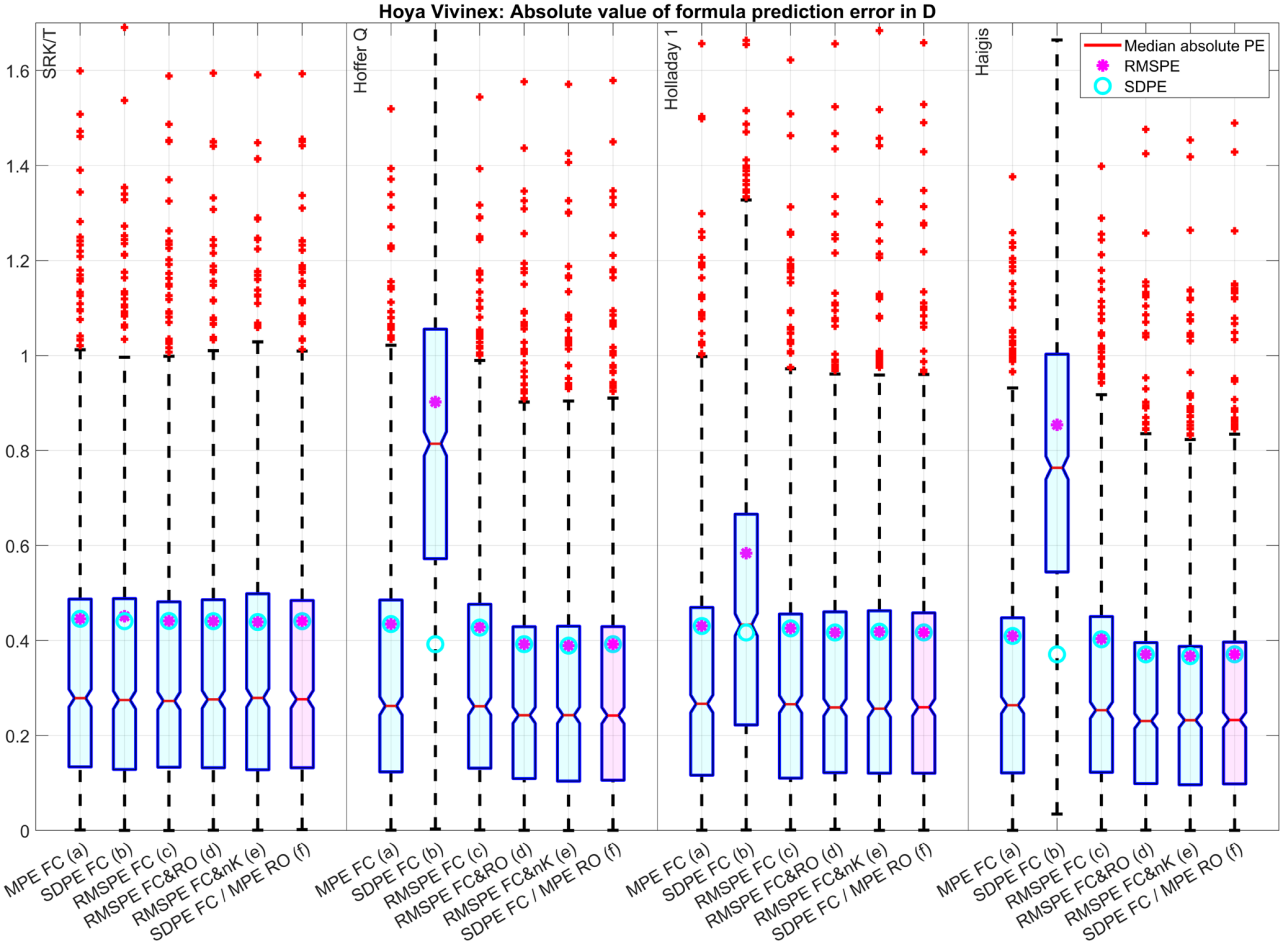
Dataset 1 with the Hoya Vivinex lens (*N* = 886): Clustered boxplot showing the absolute value of the formula prediction error (PE) for the SRK/T, Hoffer Q, Holladay 1, and Haigis (a1 = 0.4 and a2 = 0.1) formulae. Optimization was performed for the formula constant (FC) for (a) zeroing the mean prediction error (MPE), (b) minimizing the standard deviation (SDPE) and (c) root mean squared prediction error (RMSPE), simultaneously for (d) FC and the refractive offset correction (RO) and (e) FC and the keratometer index (nK), and (f) using the two‐step approach described by Gatinel, Debellemanière, Saad, Brenner, et al. (2024) involving minimizing the SDPE with FC and zeroing the MPE with RO. The lower and upper margins of the boxes refer to the quartiles, and the whiskers to the limits of the 95% confidence intervals of the absolute formula prediction error.

**FIGURE 2:**
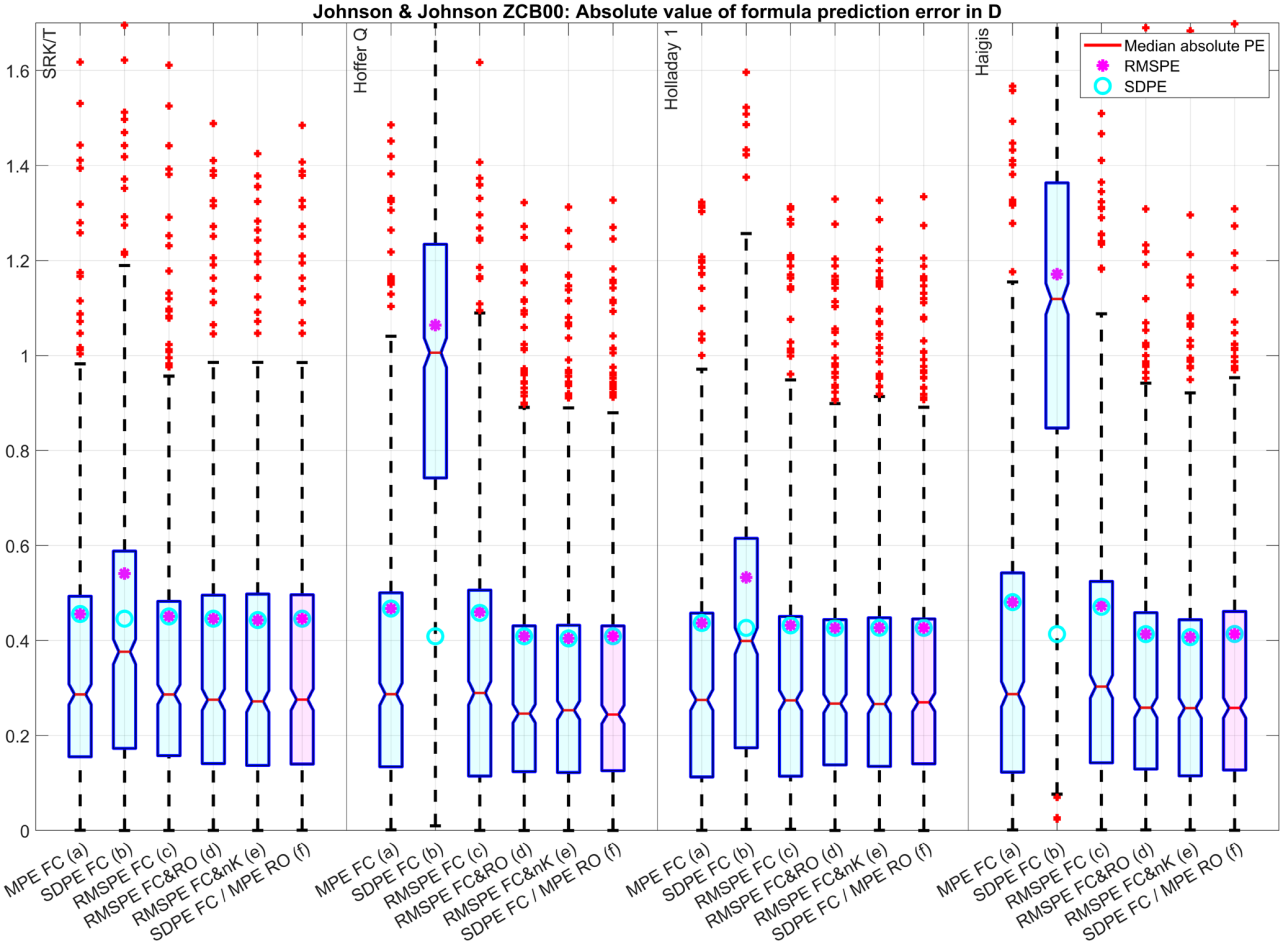
Dataset 2 with the Johnson & Johnson ZCB00 lens (*N* = 613): Clustered boxplot showing the absolute value of the formula prediction error for the SRK/T, Hoffer Q, Holladay 1, and Haigis (a1 = 0.4 and a2 = 0.1) formulae. Optimization was performed for the formula constant (FC) for (a) zeroing the mean prediction error (MPE), (b) minimizing the standard deviation (SDPE) and (c) root mean squared prediction error (RMSPE), simultaneously for (d) FC and the refractive offset correction (RO) and (e) FC and the keratometer index (nK), and (f) using the two step approach described by Gatinel, Debellemanière, Saad, Brenner, et al. (2024) involving minimizing the SDPE with FC and zeroing the MPE with RO. The lower and upper margins of the boxes refer to the quartiles, and the whiskers to the limits of the 95% confidence intervals of the absolute formula prediction error.

**FIGURE 3:**
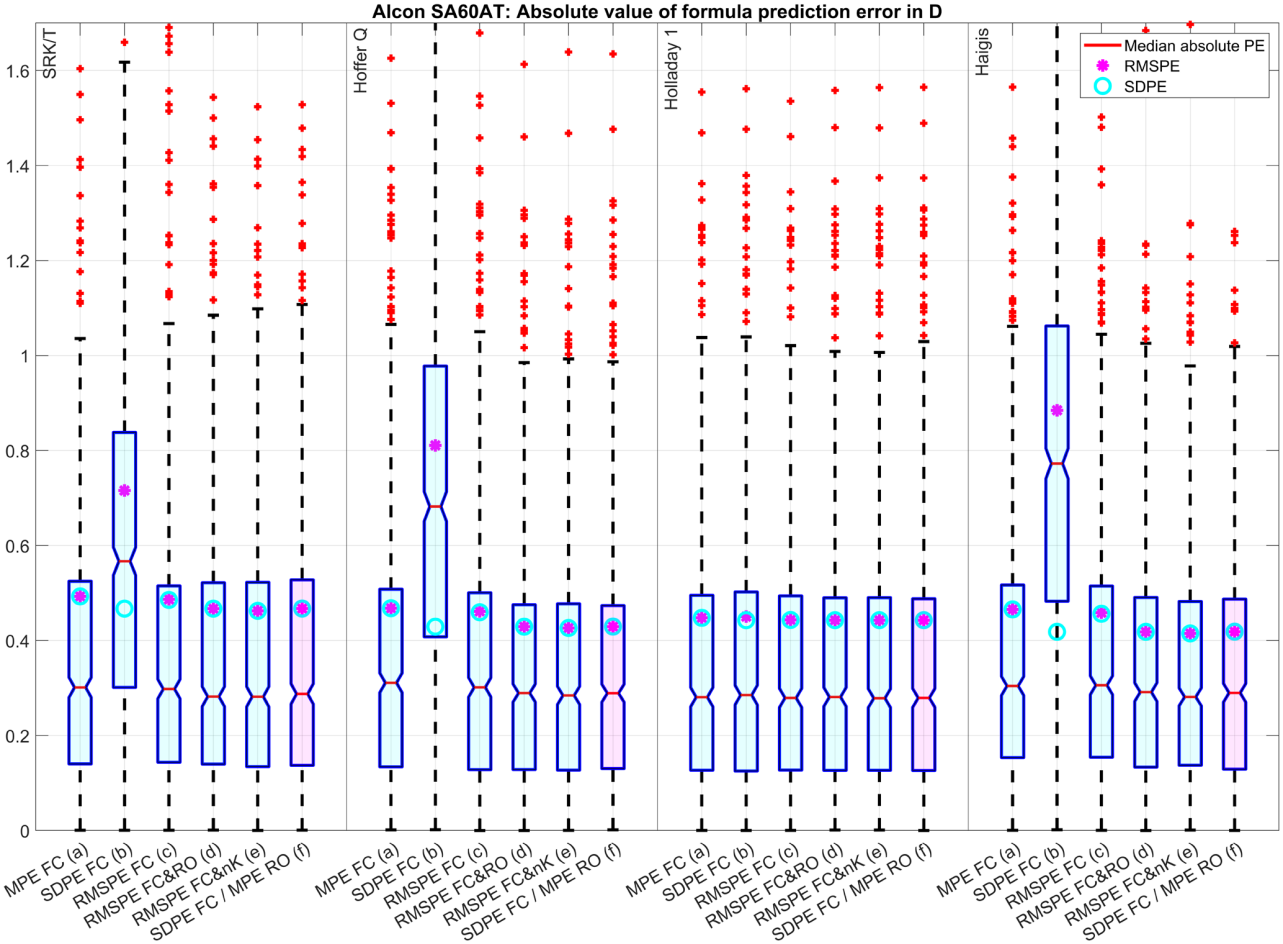
Dataset 3 with the Alcon SA60AT lens (*N* = 821): Clustered boxplot showing the absolute value of the formula prediction error for the SRK/T, Hoffer Q, Holladay 1, and Haigis (a1 = 0.4 and a2 = 0.1) formulae. Optimization was performed for the formula constant (FC) for (a) zeroing the mean prediction error (MPE), (b) minimizing the standard deviation (SDPE) and (c) root mean squared prediction error (RMSPE), simultaneously for (d) FC and the refractive offset correction (RO) and (e) FC and the keratometer index (nK), and (f) using the two‐step approach described by Gatinel, Debellemanière, Saad, Brenner, et al. (2024) involving minimizing the SDPE with FC and zeroing the MPE with RO. The lower and upper margins of the boxes refer to the quartiles, and the whiskers to the limits of the 95% confidence intervals of the absolute formula prediction error.

**FIGURE 4:**
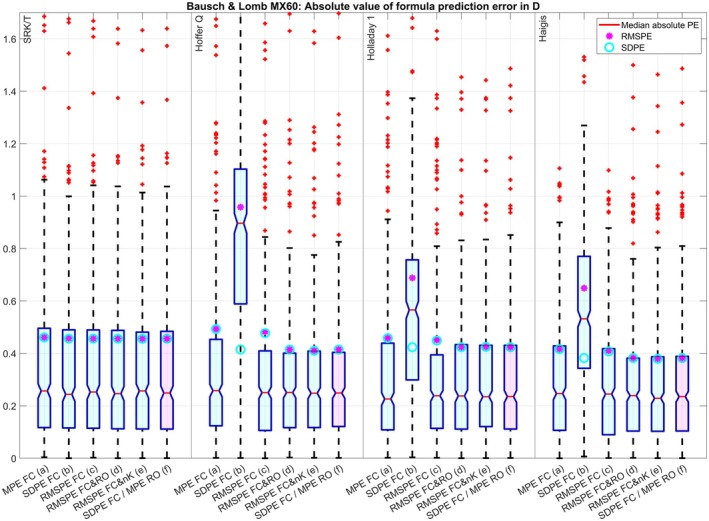
Dataset 4 with the Bausch & Lomb MX60 lens (*N* = 467): Clustered boxplot showing the absolute value of the formula prediction error for the SRK/T, Hoffer Q, Holladay 1 and Haigis (a1 = 0.4 and a2 = 0.1) formulae. Optimization was performed for the formula constant (FC) for (a) zeroing the mean prediction error (MPE), (b) minimizing the standard deviation (SDPE) and (c) root mean squared prediction error (RMSPE), simultaneously for (d) FC and the refractive offset correction (RO) and (e) FC and the keratometer index (nK), and (f) using the two‐step approach described by Gatinel, Debellemanière, Saad, Brenner, et al. (2024) involving minimizing the SDPE with FC and zeroing the MPE with RO. The lower and upper margins of the boxes refer to the quartiles, and the whiskers to the limits of the 95% confidence intervals of the absolute formula prediction error.

The clustered boxplot in Figure 1 displays the absolute value of the PE for the four formulae under test and the optimization strategies (a) to (f) for dataset 1. Except for the optimization of FC for SDPE (b) where the lower scatter in the PE (SDPE, cyan circles) is at the cost of a systematic refractive offset (indicated by the red lines (median absolute PE) or the large RMSPE (magenta dots)), the SDPE and the RMSPE match pretty well for all optimizations and all formulae indicating no systematic refractive offset. Again, with the SRK/T and Holladay formulae, optimizing only FC for zero MPE (a) or minimal RMSPE (c) seems to be sufficient whereas the Hoffer Q and the Haigis formulae seem to benefit from an optimization of FC and RO (either (d) or (f)) or an optimization of FC and nK (e) where the mean absolute PR (red lines) and the SDPE (cyan circles) and RMSPE (magenta dots) could be reduced systematically compared to optimization of FC for zero MPE (a) or minimal RMSPE (c). For dataset 2 (Figure 2) the optimization of FC for minimal SDPE (b) is not that much different to the optimizations (a), (c), (d), (e) and (f) for the SRK/T and Holladay 1 formula, but otherwise the results are comparable to those of dataset 1. For dataset 3 (Figure 3) the optimization of FC for minimal SDPE (b) is surprisingly similar to the optimizations (a), (c), (d), (e) and (f) for the Holladay 1 formula, but otherwise the results are comparable to those of dataset 1. For dataset 4 (Figure 4) the situation is quite similar to dataset 3, but here the optimization of FC for minimal SDPE (b) is surprisingly similar to the optimizations (a), (c), (d), (e) and (f) for the SRK/T formula instead of the Holladay 1 formula, but otherwise the results are comparable to those of dataset 1.

The optimisation for RMSPE with a FC and R0 (which includes zeroing of MPE and minimizing SDPE) using nonlinear iterative optimization techniques in (d) gives quite similar results to the two‐step approach (f) (which sequentially minimizes SDPE with FC and MPE with R0) for all formulae and all datasets. This is shown by the (signed) prediction error in Table 3 and the absolute value of the prediction error in Figures 1, 2, 3, 4.

## 4 DISCUSSION

### 4.1 Formula constant optimization in general

Correct and accurate formula constants are crucial for the performance of IOL power calculations in cataract surgery. However, there are no common standards or guidelines on how to optimize formula constants (Aristodemou et al., 2011; El‐Khayat & Tesha, 2021; Galvis et al., 2013; Gatinel, Debellemanière, Saad, Rampat, et al., 2023; Zhang et al., 2019). Nonlinear iterative strategies for constant optimization were a large step forward because they allowed optimization for the formula prediction error as a target variable for any metric and they were not restricted to single constant formulae (Langenbucher et al., 2022; Langenbucher, Szentmáry, Cayless, Müller, et al., 2021). It has been shown in previous papers that constants for single or triple constant formulae could be effectively optimized using nonlinear iterative optimization aimed at zeroing the mean or median PE or minimizing the mean or median absolute PE or the SDPE or RMSPE (Langenbucher et al., 2022; Langenbucher, Hoffmann, et al., 2023; Langenbucher, Szentmáry, et al., 2023; Langenbucher, Szentmáry, Cayless, Müller, et al., 2021; Langenbucher, Szentmáry, Cayless, Weisensee, et al., 2021). However, the implementation of those optimization strategies requires more involved programming, and standard applications are not available in consumer software tools such as Excel.

### 4.2 The new concept of a two‐step approach for formula constant optimization

In Gatinel, Debellemanière, Saad, Rampat, et al. (2023) developed a simple straightforward concept for formula constant optimization based on gradient descent which could be used for disclosed or non‐disclosed formulae with a single constant. This concept requires the arithmetic mean of the keratometric power and power of the implanted IOL together with a start value for the formula constant and the corresponding formula prediction error (Zhang et al., 2019). A correction term can then be derived for the formula constant for update. We were able to show that the results are surprisingly effective even after one iteration or update cycle and that the resulting MPE is close to zero. In a second paper Gatinel, Debellmaniére, Saad, Rampat, et al. (2024) generalized this strategy to optimize single constant formulae for minimal SDPE and RMSPE, and again the strategy seems to be very efficient with surprisingly good results after only a single iteration. However, what we learned from the literature is that due to the internal architecture of the formulae mostly involving tuning the ELP during constant optimization, the scatter of the PE (SDPE) is not necessarily linked to the systematic offset (MPE) (Gatinel, Debellemanière, Saad, Wallerstein, et al., 2023; Langenbucher et al., 2022; Langenbucher, Szentmáry, Cayless, Müller, et al., 2021). This means that an optimization for SDPE could yield an offset in refraction, or vice versa an optimization for zero MPE does not necessarily yield the lowest SDPE value. Optimizing for RMSPE as performed for all IOLs and formulae as a routine, for example, with IOLCon (www.iolcon.org) seems to be a good compromise as this metric includes both the arithmetic mean and the scatter of the PE (Gatinel, Debellemanière, Saad, Brenner, et al., 2024).

However, optimizing both the precision of the formula (with a low SDPE) and the accuracy (MPE close to zero) requires 2 degrees of freedom in our formulae. This means that an additional parameter which can be tuned independently of the ELP is required in addition to the FC. In the Castrop formula as described in 2021 (Langenbucher, Szentmáry, Cayless, Weisensee, et al., 2021), a constant triplet is used where 2 of the constants tune the ELP and another constant is dedicated to considering an offset in refraction (which is mostly equivalent to a shift in keratometric power). In a recent paper from Gatinel, Debellemanière, Saad, Brenner, et al. (2024) demonstrated a new strategy with a straightforward optimization of FC for SDPE (which could lead to a systematic offset in MPE) and subsequently a correction of this refractive offset RO to shift the PE distribution to zero the arithmetic mean. The first step of the two‐step approach is equivalent to the previous paper (Gatinel, Debellmaniére, Saad, Rampat, et al., 2024), and the second step is a simple offset correction (Gatinel, Debellemanière, Saad, Brenner, et al., 2024; Gatinel, Debellemanière, Saad, Rampat, et al., 2023). However, because the RO varies between IOL models, it could not be integrated in the formula as a fixed value. Instead, it was treated as a second formula constant similar to the R constant of the Castrop formula (Langenbucher, Szentmáry, Cayless, Weisensee, et al., 2021).

### 4.3 Our study setting and clinical implications

In the present paper, we implemented the optimization strategy described by Gatinel, Debellemanière, Saad, Brenner, et al. (2024) and compared the results to known nonlinear iterative constant optimization techniques which have been validated before with several datasets and formulae. Based on four datasets with modern hydrophobic aberration correcting IOL (Hoya Vivinex and Johnson & Johnson ZCB00), a spherical IOL (Alcon SA60AT) and an aberration‐free IOL (Bausch & Lomb MX60), we optimised the FC for four classical third and fourth generation formulae for zero MPE, for minimal SDPE, and for minimal RMSPE. In addition, to allow a direct comparison to the strategy described by Gatinel, Debellemanière, Saad, Brenner, et al. (2024) (Langenbucher et al., 2024) we implemented a nonlinear iterative optimization which minimized RMSPE for both the FC and RO. To get some idea as to whether a refractive offset correction (equivalent to an offset in keratometric power) or a tuning of nK (which is equivalent to a scaling of keratometric power) is more efficient, we also implemented nonlinear iterative optimization which minimized RMSPE for both the FC and nK.

Our results confirm our findings from previous studies that optimizing FC for minimal SDPE only is in general not a good idea (Langenbucher et al., 2022; Langenbucher, Hoffmann, et al., 2023; Langenbucher, Szentmáry, et al., 2023; Langenbucher, Szentmáry, Cayless, Müller, et al., 2021), since the good precision of the formula (small SDPE) is at the cost of a systematic offset in the refraction (MPE) (Langenbucher, Szentmáry, Cayless, Müller, et al., 2021). Optimizing FC for zero MPE or RMSPE did not make a clinically relevant difference for all datasets and formulae. If we allow RO or nK as a ‘second formula constant’ then the Hoffer Q and the Haigis formulae mostly performed systematically better for all datasets, whereas the SRK/T or Holladay 1 formulae showed only a slight or no improvement. In addition, we found that there is no clinically relevant difference if we use RO or nK as a ‘second formula constant’. Therefore, as we feel that it is more straightforward to use RO instead of an individual nK for each optimization, especially in the case of the Hoffer Q and the Haigis formulae we recommend optimizing and using a constant doublet consisting of pACD and RO or a0 and RO. The two‐step approach of Gatinel, Debellemanière, Saad, Brenner, et al. (2024) (f) yielded surprisingly good results for all formulae under test and all four datasets. While the nonlinear optimization process, which can be seen in this context as the gold standard, will always intrinsically match or exceed the performance of the two‐step process, the two‐step process has the advantage of efficiency and the differences are small. In fact, under the conditions examined here, there is no clinically relevant difference as compared to the nonlinear iterative optimization for FC and RO (d), making the two‐step process a viable alternative. The differences in the RMSPE between both methods are typically in the 3rd decimal place only.

## 5 LIMITATIONS

However, our study shows some limitations: (1) we restricted the study to four classical fully disclosed formulae of the third and fourth generation. With modern formulae which use more realistic keratometer indices (Hoffer & Savini, 2020; Savini et al., 2020) the differences in the formula constants between (a) or (c) and (b) are expected to be smaller. (2) The results presented here are based on four monocentric datasets with only 2787 subjects in total. These results should be validated against large prospective multicentric datasets and for other IOL models. (3) Implementing an additional tuning parameter (RO or nK) for IOL power formulae has to be discussed with the formula authors first as it directly affects the architecture of the formula. And (4) the benefit of using a second tuning parameter should be validated using a rigorous crossvalidation with the dataset split into training and test data or, as a minimum, with a k‐fold crossvalidation or bootstrap/Jackknife resampling technique.

## 6 FUTURE WORK

Further multicentric prospective studies with other intraocular lens models and larger sample sizes should be carried out to completely assess and validate this novel two‐step approach. A rigorous cross‐validation would allow for an unbiased evaluation of the results and a comparison to standard strategies of formula constant optimisation. If the authors of undisclosed lens formulae could be motivated to provide macros for standard consumer software such as Excel or WEB API to predict spectacle refraction from biometric data, the power of the implanted lens, and preset formula constant(s) the performance of this two‐step approach could be evaluated and compared to legacy optimisation techniques and also applied to modern formulae (such as Barrett Universal, Kane, EVO, K6, Olsen, 2006).

In conclusion, this paper shows that using a second tuning parameter which does not interact with the effective intraocular lens position in addition to the formula constant could systematically improve the performance of classical intraocular lens power formulae. With a refractive offset correction or tuning the keratometer index especially the Hoffer Q and the Haigis formulae yield systematically better results in terms of precision and accuracy. This concept involving a second tuning parameter could be implemented using nonlinear iterative optimization strategies or alternatively with a two‐step approach according to Gatinel, Debellemanière, Saad, Brenner, et al. (2024) involving first minimizing the standard deviation and then zeroing the mean prediction error. Both techniques are equivalent in terms of mean, standard deviation, and root mean squared prediction error. Where nonlinear iterative optimization is not available or too complex, this two‐step approach offers greater efficiency, making it a very good alternative which could easily be implemented even in consumer software tools such as Excel.

## CONFLICT OF INTEREST STATEMENT

Dr. Cayless, Dr. Gatinel, and Dr. Szentmáry do not report any financial interests. Dr. Hoffmann reports speaker fees from Heidelberg engineering, Hoya Surgical, and Johnson & Johnson outside the submitted work. Dr. Langenbucher reports speaker fees from Hoya Surgical and Johnson & Johnson Vision outside the submitted work. Dr. Wendelstein reports speaker fees from Carl Zeiss Meditec AG, Rayner, Alcon, and Johnson & Johnson Vision outside of the submitted work.

## ETHICS STATEMENT

The data analysed in this retrospective study were transferred to us in an anonymized fashion, which precludes back‐tracing of the patient. The local Institutional Review Board (Ärztekammer des Saarlandes, registration number 157/21) provided a waiver for this study, and patient informed consent was not required for this study.
